# Editorial overview of Pearls Microbiome Series: E pluribus unum

**DOI:** 10.1371/journal.ppat.1009912

**Published:** 2021-08-31

**Authors:** Deborah A. Hogan, Joseph Heitman, Mary Ann Jabra-Rizk, Laura J. Knoll, John M. Leong, Neal Silverman

**Affiliations:** 1 Department of Microbiology and Immunology, Geisel School of Medicine at Dartmouth, Hanover, New Hampshire, United States of America; 2 Departments of Molecular Genetics and Microbiology (MGM), Pharmacology and Cancer Biology, and Medicine, Duke University Medical Center, Durham, North Carolina, United States of America; 3 Department of Oncology and Diagnostic Sciences, Dental School, University of Maryland, Baltimore, Maryland, United States of America; 4 Department of Microbiology and Immunology, School of Medicine, University of Maryland, Baltimore, Maryland, United States of America; 5 Department of Medical Microbiology and Immunology, University of Wisconsin–Madison, Madison, Wisconsin, United States of America; 6 Department of Molecular Biology and Microbiology, Tufts University School of Medicine, Boston, Massachusetts, United States of America; 7 Department of Medicine, Division of Infectious Diseases, University of Massachusetts Medical School, Worcester, Massachusetts, United States of America

## Introduction

The human microbiome constitutes the collection of all the microorganisms living in association with the human body with each body site being home to a unique microbial community [1]. Human-associated microbial communities can include eukaryotes, archaea, bacteria, and viruses and provide protection against foreign invaders, stimulate the immune response, produce antimicrobials, and aid in digestion among other functions. Our understanding of the link between the human microbiome and disease is rapidly expanding in large part due to revolutionizing advances in next generation sequencing. In fact, an ever-growing number of studies have demonstrated that changes in the composition of our microbiomes correlate with numerous disease states or responses to treatment. However, understanding the impact of shifts in microbial communities on health and disease and the mechanisms that confer stability in the microbiome have been challenging to elucidate, due to the vast microbial diversity and differences between individuals. Nevertheless, the notion that manipulation of microbial communities may provide prophylactic or therapeutic tools to improve human health has been the focus of much research [2]. Here, we highlight a collection of Pearls articles delving into the current state of knowledge linking the microbiome to human disease.

## The fungal microbiome—An overlooked kingdom of the microbiome

Despite the considerable evaluations of bacterial communities, fungal constituents of the microbiome, termed the “mycobiome,” have received much less attention. Yet increasing evidence from studies in this emerging field have indicated that fungi are central to maintaining gut and oral homeostasis and systemic immunity [3–6], highlighting the need for more comprehensive mycobiome characterization. In this collection of Pearls, 7 articles addressed the role of fungi in health and disease at multiple body sites, namely the oral cavity, lungs, skin, and the gastrointestinal tract (GI). Two Pearls tackled the oral microbiome, an exceptionally complex ecosystem harboring diverse microbial communities. Combined, the 2 articles presented findings from animal and clinical studies demonstrating that perturbations that disrupt the oral microbial equilibrium may lead to the development of oral disease with a specific focus on the clinical implications of fungal–bacterial interactions. An overview of the oral microbiome was presented in a Pearl by Sultan and colleagues [7] where it was stressed that unlike the oral bacteriome, the mycobiome in general is a new and poorly recognized biome. In the article, the authors describe the interactions between the fungal pathogen *Candida albicans* and the streptococci, as these diverse microbial species co-colonize the oral cavity. These fungal–bacterial interactions are largely considered synergistic, where the bacteria provide adhesion sites for the fungus to colonize the oral cavity, as well as a carbon source for growth [8]. In return, by utilizing the lactic acid produced by streptococci, *C*. *albicans* lowers the oxygen tension levels, which is advantageous to the bacteria [9]. This mutualistic fungal–bacterial relationship may, however, have repercussions to the host, as was demonstrated by animal studies indicating that the interactions between *C*. *albicans* and the cariogenic bacterial species *Streptococcus mutans* may impact the development of dental caries (tooth decay) [10]. This specific interspecies interaction was further dissected in a Pearl by Koo and colleagues [11], where the authors elaborate that by enhancing bacterial colonization and biofilm formation on oral surfaces, *C*. *albicans* becomes more invasive, exacerbating mucosal tissue infection and destruction [12]. Furthermore, the physical co-adhesion between *C*. *albicans* with streptococci mediated by surface adhesins and receptors on both organisms increases tooth surface colonization and enhances microbial burden, promoting biofilm formation on tooth surface [13]. Interestingly, the bacteria were also shown to promote hyphal formation and expression of proteolytic enzymes, key virulence attributes in *C*. *albicans*, further facilitating tissue invasion and inflammatory processes. By summarizing findings from clinical and animal studies, the Pearl by Koo and colleagues underscores the importance of fungal–bacterial interactions in the severity of oral mucosal diseases and dental caries. Collectively, the Pearls by Sultan and colleagues [7] and Koo and colleagues [11] clearly establish fungi as a significant component of the oral microbiome.

The GI, which extends from the oral cavity, houses the largest and most diverse populations of microorganisms within the human body [14]. Several studies in recent years detailing the importance of fungi in the human gut have highlighted the need for more comprehensive investigations into the contributions of the intestinal mycobiome to health and disease. Galloway-Peña and Kontoyiannis [15] focused on the role of the gut mycobiome in different patient populations and the importance of bacterial–fungal dysbiosis in inflammatory GI disorders, including Crohn disease and ulcerative colitis, as well as in the pathogenesis of colon adenomas and pancreatic ductal adenocarcinoma as discussed further below. Lastly, the immunomodulatory role of the gut mycobiota in both innate and adaptive immunity was also highlighted. The role of fungi in intestinal inflammation was also the topic in the article by Wong and colleagues [16] within the context of microbes in inflammatory bowel disease (IBD), which the authors state could be explained by an aberrant response to the gut microbial community. In one described study, mice with the yeast *Saccharomyces cerevisiae* were shown to promote colitis via enhanced purine metabolism [17]; although the contribution of *S*. *cerevisiae* remains unclear, a role for the fungal communities in the gut in IBD is supported by studies examining the innate immune receptor for fungal glucans Dectin-1 [18].

Although the oral and gut mycobiome are the best studied, an interesting Pearl by Hamm and colleagues [19] discussed the lung microbiome; in the article, the authors propose that the lung mycobiome may form a reservoir for opportunistic fungal mycoses caused by commensal lung-adapted fungi. In regard to the skin mycobiome, a Pearl by Findley and Grice [20] described a study identifying *Malassezia* as the most abundant skin fungal colonizer, which, although a skin commensal, is associated with atopic dermatitis, tinea versicolor, dandruff, and psoriasis. In addition to *Malassezia*, other skin fungal species such as *Trichophyton* and *Candida* species have also been linked to dermatological disorders. The majority of the articles in the microbiome series dealt with the human microbiome and human diseases; however, an intriguing Pearl by Vannier and colleagues [21] proposed that in nature, similar to humans, plants may equally rely on their resident microbiota and immune system to restrict pathogen invasion; in fact, microbiota-mediated disease resistance could confer extended immune functions to the plant host. A case in point, the authors describe a recent study demonstrating that the presence of coresident bacteria in the roots of the plant *Arabidopsis thaliana* colonized by deleterious filamentous eukaryotes maintains fungal balance in plant roots and, in turn, the health of the plants [22].

Recent advances in molecular biology have facilitated analyses of the human microbiome, yet we do not understand a great deal about the processes underlying the transition from a healthy to a disease-associated microbiome, and, specifically, the role of fungi in propagating disease. Combined, the presented 7 articles provide crucial insights into the important role of fungi as a component of the human microbiome and argue that the mycobiome should not be excluded from future oral microbiome studies. Specifically, additional research is warranted to understand the multifaceted interactions between fungi and the host, as well as between fungi and other microbial species.

## Bacterial microbiome Pearls

Over the last 5 years, 13 Pearls on the bacterial microbiome have been published as part of this series. Several articles focus on microbial genetics and newly discovered species. For example, Carrow and colleagues [23] drill down on the implications of immense genetic heterogeneity within a single species of common gut commensal bacteria, *Bacteroides fragilis*, which is known to promote immune tolerance in the gut but can be inflammatory in other contexts. Two Pearls, one by Rowley and Kendall [24] and one by Putnam and Goodman [25], focus on the uptake of B vitamins, in particular thiamine (B1) and cobalamin (B12), and the mechanisms used by different members of the gut microbiome community to compete or cooperate for these limiting resources. Vitamin A, which has profound effects on animal development as while as immunity, is the focus of Iyer and Vaishnava [26]; acute phase responses decrease the transport of retinols to the liver and mobilizes vitamin A activity locally in the gut, contributing to T helper 17 (Th17) responses in addition effects of microbes directly.

Other Pearls, including the one by Ludington and Ja [27], discussed microbiome studies performed using model laboratory systems such as *Drosophila*. This model has a less complex microbial community, compared to mammals, which is partly sustained in standard laboratory conditions through fecal shedding, growth in fly media, and re-ingestion. This article reviews earlier studies highlighting the effect of the microbiome on fly behavior, life span, and developmental growth. Additionally, recent elegant approaches that have been used to differentiate passenger microbes from those that truly establish colonization and growth within the insect gut are also described. These tools can be used to dissect how these various microbes generate their effects on animal physiology using a model with less complex communities but still robust intra-kingdom communication and competition.

Several other Pearls focus on mice and other vertebrate models of host–microbiome interactions. Aleman and Valenzano [28] describe how the microbial communities in the gut change as a function of aging, potentially contributing to the aging process(es). Using killifish, which are a facile vertebrate model for aging due to their short life span, transfer of young microbiota into middle-aged animals extended life span. The effect of immunity on the microbiome, and vice versa, was the subject of 4 Pearls; Roland and colleagues [29] review how polymorphisms in the human leukocyte antigen (HLA)/major histocompatibility complex (MHC) locus affects healthy commensal communities through education of appropriate regulatory T cell (Treg) and Th17 development in the gut. Wilson and colleagues [30], on the other hand, described how microbial changes in the airways contribute to asthma, by altering the activity of numerous immune cell types. Interestingly, Gilbert and Lewis [2] presented the “covert pathogenesis” concept, whereby a bug may have a hit-and-run type of influence on a later pathogenic event and how the ability of a microbe to cause this type of damage is influenced by the microbiota in the local tissue. Another concept discussed in the Pearl by Litvak and Baumler [31] is that of colonization resistance; here, once a niche is filled by a microbe, for example, when acquired during early neonatal life, that niche becomes resistant to colonization by other microbes, in particular pathogens. In their discussion, the authors focus on the interindividual variation in microbial communities that cannot be explained solely by differences in genetics or diet. Rather, they posit that colonization order, early in life, is key to setting up these stable microbial communities.

The microbiome and the various ways it can affect human physiology through small molecules was the topic of Pearl by Glowacki and Martens [32]. The described effects include interaction with and metabolism of drugs creating toxic metabolites, the ability of commensals to de novo generate toxins including alcohol (in auto-brewery syndrome) or other toxins, by liberating immunogenic peptides, by generating bioactive chemicals, or by releasing immune modulation macromolecules. These molecules can affect multiple different organ systems, including locally within the gut, as well as at more distant locations such as the brain and the cardiovascular system. Foley and colleagues [33] focus specifically on one class of compounds generated by the gut microbiome, the secondary bile acids. The microbiome can transform bile acids, a product of the liver, into a variety of potentially bioactive compounds known as secondary bile acids. The first required step in secondary bile acid production involves microbial enzymes known as bile salt hydrolases, which de-conjugate bile acids, making them available for secondary modification by a variety of microbiota-derived enzymes. These secondary bile acids enter circulation and signal via multiple receptors including farnesoid X receptor (FXR), other nuclear receptors, as well as G protein–coupled receptors (GPCRs).

## Parasites and the microbiome

Few Pearls have been written on eukaryotic parasites in the microbiome, collectively referred to as the parasitome, mainly because few studies have focused on the role that parasites play in a healthy microbiome. This dearth of information was discussed during the first Parasite Microbiome Project Workshop, and a grand challenge was written by conference attendees [34]. Identification of parasites in the microbiome is complicated by the fact that they are eukaryotes, and sequencing 18S ribosomal RNA provides largely host sequences. Additionally, most parasites have complex life cycles with multiple stages that are difficult or not yet possible to mimic in culture conditions. Another limitation for these studies is that many parasites have their own microbiome with viral or bacterial symbionts [34].

Historically, protists and helminths have been considered parasites because of their negative impact on the host, but emerging evidence suggests that protists are common residents of the mammalian gut, including humans, and often have commensal or beneficial relationships with the host [35]. While *Blastocystis hominis* and *Dientamoeba fragilis* are associated in the literature with gastrointestinal disease, they are actually at a higher prevalence in healthy individuals than diseased ones. The loss of diversity in the parasitome of the gut has been correlated with the rapid increase in autoimmune and inflammatory diseases [35]. For IBD, specifically, there were noted protective effects from colonization with *Tritrichomonas* species [16]. As many intestinal protists, such as *Entamoeba* spp., *Endolimax nana*, and *Iodamoeba butschlii*, are amoeba that can phagocytize bacteria, it follows logically that colonization with them will change the composition of the bacteriome. The most studied is the global pathogen *Entamoeba histolytica*, for which the bacteriome can provide nutrition after phagocytosis as well as serve as an immune response trainer, a first line of defense against colonization, or processor of metabolites that can be available as nutrition or inhibit parasite encystation [36]. The positive and negative effects of nonpathogenic amoeba interactions with bacteria have not yet been examined.

## Viruses in the microbiome

Like fungi and parasites, viruses are underappreciated for their roles in microbiome communities. Four Pearls focus specifically on eukaryotic and bacterial viruses in the microbiome, referred to as the virome. A Pearl contributed by Wang [37] discusses progress in characterizing viromes and the increasing recognition of the importance of different viruses in the context of diverse clinical states such as HIV infection, IBD, malnutrition, graft-versus-host disease, and type 1 diabetes. Challenges to defining the virome include the need for improved reference databases, sampling methods that capture both DNA and RNA viruses, and an enhanced spectrum of viral culture methodologies, and strong arguments are made for increased leveraging of commonalities in the study of bacterial and eukaryotic viruses [37].

Duerkop [38] specifically focuses on phages within the mammalian microbiota and how these viruses directly and indirectly influence microbial communities and host–microbe interactions. Importantly, the recent studies included in this work highlight the differences in phage communities observed between family members, upon diet changes, and after antibiotic treatment independent of changes in the bacterial microbiome, and these dynamics suggest functional roles for viruses.

Two Pearls highlight how microbiome members can affect the course of viral infections and how viruses affect bacterial virulence and clearance. A Pearl by Sullender and Baldridge [39] describes data indicating that commensal bacteria and parasites contribute to infections by enteric viruses including human norovirus (HNoV), a major cause of acute diarrhea worldwide. The means by which microbiome organisms promote infection is multifactorial and includes physical interactions, effects on immune signaling, and the induction of specific intestinal cell types that are targeted by the virus [39]. Neu and Mainou [40] discuss the mechanisms by which eukaryotic and bacterial viruses shape microbial communities directly through physical interactions. For example, viruses such as influenza A and respiratory syncytial virus (RSV) directly bind specific gram-positive and/or gram-negative bacteria, thereby affecting macrophage uptake and epithelial adherence; these interactions also promote virion stability. At the same time, bacterial products may contribute to virus inactivation.

## Cancer and the microbiome

The growing appreciation of the powerful effects of organisms within the microbiome on inflammation and immunity, cell proliferation, and chemical resistance has stimulated research on the roles of the microbiome in cancer etiology and treatment. Clinical studies suggest that diverse cancers of the alimentary tract, female reproductive tract, and lymphoma are influenced by microbiomes, and, over the past 3 years, *PLOS Pathogens* has published 5 Pearls on these topics. These articles build on a previous *PLOS Pathogens* collection entitled “What we can learn about infectious disease and cancer,” which was comprised of 18 Pearls that highlighted diverse ways in which microbes contribute to cancers and affected cancer treatments [41].

One of the best studied examples of a microbe as a carcinogen is *Helicobacter pylori* in the context of gastric cancer, and a Pearl by Butt and Epplein [42] focused on the potential relationships between *H*. *pylori* and other cancers, namely colorectal cancer (CRC). The authors discuss the ways that *H*. *pylori*, a bacterium that is primarily localized to the stomach, may influence CRC through effects on the intestinal microbiome, host endocrine signaling, metabolism, or inflammation. A piece by Ajayi and colleagues [43] highlights new work on the connection between the microbiome and Barrett esophagus (BE), in which normal squamous epithelial cells differentiate to a state that is linked to esophageal adenocarcinomas. Recent molecular, culture-based, and histological studies found differences in associated microbes between samples associated with BE or esophageal adenocarcinomas relative to comparator samples with lower levels of gram-positive streptococci and higher levels of gram-negative bacteria (e.g., *Escherichia coli*, *Campylobacter*, and *Fusobacterium*) associated with disease. While the ways by which esophageal microbiota contributes to BE and esophageal adenocarcinoma are not yet known, hypotheses include effects on local inflammation.

Dheilly and colleagues [44] extend the concept of the indirect effects of microbes on cancers by highlighting processes that are known to be modulated by pathogens (angiogenesis, host cell death, host cell replication control, etc.), which can also impact the proliferation of cancerous cells. Relationships between viruses and parasites are the focus of this review. For example, immune suppression induced by the parasite *Plasmodium falciparum* allows Epstein–Barr virus (EBV)-infected B cells to proliferate in Burkitt lymphoma. Similarly, the parasitic nematode *Strongyloides stercoralis* hastens the development of cancer in human T-lymphotropic virus type 1 (HTLV-1)-infected cells, *Trichomonas vaginalis* is linked to cervical cancer, and *Schistosoma haematobium*, a urinary blood fluke, is a risk factor for squamous cell carcinoma of the bladder. Furthermore, parasites can harbor other microbes in their own microbiomes, which can provide protection and increase the spread of organisms that are oncogenic or that attenuate native immune mechanisms that suppress the persistence and spread of cancerous cells. The complex interplay between the host, viruses, and other microbes is also the focus of Lin and colleagues [45], which describes factors that influence the development of invasive cervical cancer driven by human papillomavirus (HPV). Data indicate that HPV alone does not lead to the development of cancer; other factors in the mucosal environment such as epithelial integrity, mucus, immune regulation, and the local microbiota also contribute. As in the case of esophageal adenocarcinoma [43], invasive cervical cancer was associated with the decrease of beneficial microbiome members (e.g., *Lactobacilli*) and increased levels of gram-negative species such as *Fusobacterium*. Furthermore, Neu and Mainou [40] summarize recent studies on ways that bacteria can promote fitness of diverse virus types by enhancing virion stability, promoting infection of eukaryotic cells.

There is growing interest in the effects of the microbiome on the response to cancer therapies [45], and this important topic was the focus in a piece by Galloway-Peña and Kontoyiannis [15]. It is now appreciated that bacteria in the microbiome affect treatment-related toxicities and transplant outcomes. For example, *Candida* spp. are strongly implicated in graft-versus-host disease, a condition in which donated bone marrow or peripheral blood stem cells attack the recipient’s body. Thus, it is important to assess how potential therapies affect fungal species composition and levels in the gastrointestinal microbiome and if changes in the mycobiome impact outcomes.

## Conclusions

Several common themes across the Pearls on the microbiome emerged including the need to characterize the complete microbiome (bacteria, fungi, parasites, and bacterial and eukaryotic viruses), the value of longitudinal studies, and the need for robust nondisease reference groups [42,43]. Galloway-Peña and Kontoyiannis [15] also highlighted the need for diagnostic markers for clinically relevant microbiome features. Furthermore, new sampling methods such as nonendoscopic approaches may also facilitate microbiome research and diagnoses [43]. Three-dimensional (3D) cultures derived from pluripotent stem cells isolated from primary clinical samples will also provide the ability to analyze microbe–microbe host interactions and compete with innate immune responses and more specific tissue tropism, particularly, for viruses [46], but also for other microbes. The use of clinical data to inform animal and cell culture models has revealed many complex mechanisms that participate in a web of interactions that support microbiome stability and health but can also contribute to disease in either subtle or overt ways. We hope that additional *PLOS Pathogens* contributions in this area will promote cross-fertilization between microbiome subfields in ways that will hasten the discovery of unifying concepts that ultimately lead to strategies to reduce disease risk and improve treatment outcomes.

## References

[1] Dethlefsen L, McFall-Ngai M, Relman DA. An ecological and evolutionary perspective on human-microbe mutualism and disease. Nature. 2007;449(7164):811–8. Epub 2007 Oct 19. doi: 10.1038/nature06245 . 17943117 PMC9464033

[2] Gilbert NM, Lewis AL. Covert pathogenesis: Transient exposures to microbes as triggers of disease. PLoS Pathog. 2019;15(3):e1007586. Epub 2019 Mar 29. doi: 10.1371/journal.ppat.1007586; PubMed Central PMCID: PMC6438445. 30921443 PMC6438445

[3] Hernandez-Santos N, Klein BS. Through the scope darkly: the gut mycobiome comes into focus. Cell Host Microbe. 2017;22(6):728–9. Epub 2017 Dec 15. doi: 10.1016/j.chom.2017.11.013 ; PubMed Central PMCID: PMC5964996. 29241038 PMC5964996

[4] Ghannoum MA, Jurevic RJ, Mukherjee PK, Cui F, Sikaroodi M, Naqvi A, et al. Characterization of the oral fungal microbiome (mycobiome) in healthy individuals. PLoS Pathog. 2010;6(1):e1000713. Epub 2010 Jan 15. doi: 10.1371/journal.ppat.1000713; PubMed Central PMCID: PMC2795202. 20072605 PMC2795202

[5] Hallen-Adams HE, Suhr MJ. Fungi in the healthy human gastrointestinal tract. Virulence. 2017;8(3):352–8. Epub 2016 Oct 30. doi: 10.1080/21505594.2016.1247140 ; PubMed Central PMCID: PMC5411236. 27736307 PMC5411236

[6] Li J, Chen D, Yu B, He J, Zheng P, Mao X, et al. Fungi in gastrointestinal tracts of human and mice: from community to functions. Microb Ecol. 2018;75(4):821–9. Epub 2017 Nov 8. doi: 10.1007/s00248-017-1105-9 . 29110065

[7] Sultan AS, Kong EF, Rizk AM, Jabra-Rizk MA. The oral microbiome: A Lesson in coexistence. PLoS Pathog. 2018;14(1):e1006719. Epub 2018 Jan 26. doi: 10.1371/journal.ppat.1006719; PubMed Central PMCID: PMC5784999. 29370304 PMC5784999

[8] Jenkinson HF, Lamont RJ. Oral microbial communities in sickness and in health. Trends Microbiol. 2005;13(12):589–95. Epub 2005 Oct 11. doi: 10.1016/j.tim.2005.09.006 . 16214341

[9] Krom BP, Kidwai S, Ten Cate JM. *Candida* and other fungal species: forgotten players of healthy oral microbiota. J Dent Res. 2014;93(5):445–51. Epub 2014 Feb 4. doi: 10.1177/0022034514521814 . 24487378

[10] Khoury ZH, Vila T, Puthran TR, Sultan AS, Montelongo-Jauregui D, Melo MAS, et al. The role of *Candida albicans* secreted polysaccharides in augmenting *Streptococcus mutans* adherence and mixed biofilm formation: In vitro and in vivo studies. Front Microbiol. 2020;11:307. Epub 2020 Apr 8. doi: 10.3389/fmicb.2020.00307; PubMed Central PMCID: PMC7093027. 32256460 PMC7093027

[11] Koo H, Andes DR, Krysan DJ. *Candida*-streptococcal interactions in biofilm-associated oral diseases. PLoS Pathog. 2018;14(12):e1007342. Epub 2018 Dec 14. doi: 10.1371/journal.ppat.1007342; PubMed Central PMCID: PMC6292568. 30543717 PMC6292568

[12] Xu H, Sobue T, Thompson A, Xie Z, Poon K, Ricker A, et al. Streptococcal co-infection augments *Candida* pathogenicity by amplifying the mucosal inflammatory response. Cell Microbiol. 2014;16(2):214–31. Epub 2013 Oct 2. doi: 10.1111/cmi.12216 ; PubMed Central PMCID: PMC3956708. 24079976 PMC3956708

[13] Falsetta ML, Klein MI, Colonne PM, Scott-Anne K, Gregoire S, Pai CH, et al. Symbiotic relationship between *Streptococcus mutans* and *Candida albicans* synergizes virulence of plaque biofilms in vivo. Infect Immun. 2014;82(5):1968–81. Epub 2014 Feb 26. doi: 10.1128/IAI.00087-14 ; PubMed Central PMCID: PMC3993459. 24566629 PMC3993459

[14] Lloyd-Price J, Mahurkar A, Rahnavard G, Crabtree J, Orvis J, Hall AB, et al. Strains, functions and dynamics in the expanded Human Microbiome Project. Nature. 2017;550(7674):61–6. Epub 2017 Sep 28. doi: 10.1038/nature23889 ; PubMed Central PMCID: PMC5831082. 28953883 PMC5831082

[15] Galloway-Peña JR, Kontoyiannis DP. The gut mycobiome: The overlooked constituent of clinical outcomes and treatment complications in patients with cancer and other immunosuppressive conditions. PLoS Pathog. 2020;16(4):e1008353. Epub 2020 Apr 3. doi: 10.1371/journal.ppat.1008353; PubMed Central PMCID: PMC7117661. 32240277 PMC7117661

[16] Wong SY, Cadwell K. There was collusion: Microbes in inflammatory bowel disease. PLoS Pathog. 2018;14(9):e1007215. Epub 2018 Sep 21. doi: 10.1371/journal.ppat.1007215; PubMed Central PMCID: PMC6147501. 30235350 PMC6147501

[17] Chiaro TR, Soto R, Zac Stephens W, Kubinak JL, Petersen C, Gogokhia L, et al. A member of the gut mycobiota modulates host purine metabolism exacerbating colitis in mice. Sci Transl Med. 2017;9(380). Epub 2017 Mar 10. doi: 10.1126/scitranslmed.aaf9044; PubMed Central PMCID: PMC5994919. 28275154 PMC5994919

[18] Iliev ID, Funari VA, Taylor KD, Nguyen Q, Reyes CN, Strom SP, et al. Interactions between commensal fungi and the C-type lectin receptor Dectin-1 influence colitis. Science. 2012;336(6086):1314–7. Epub 2012 Jun 8. doi: 10.1126/science.1221789 ; PubMed Central PMCID: PMC3432565. 22674328 PMC3432565

[19] Hamm PS, Taylor JW, Cook JA, Natvig DO. Decades-old studies of fungi associated with mammalian lungs and modern DNA sequencing approaches help define the nature of the lung mycobiome. PLoS Pathog. 2020;16(7):e1008684. Epub 2020 Jul 31. doi: 10.1371/journal.ppat.1008684; PubMed Central PMCID: PMC7392203. 32730326 PMC7392203

[20] Findley K, Grice EA. The skin microbiome: a focus on pathogens and their association with skin disease. PLoS Pathog. 2014;10(10):e1004436. Epub 2014 Nov 14. doi: 10.1371/journal.ppat.1004436; PubMed Central PMCID: PMC4231143. 25393405 PMC4231143

[21] Vannier N, Agler M, Hacquard S. Microbiota-mediated disease resistance in plants. PLoS Pathog. 2019;15(6):e1007740. Epub 2019 Jun 14. doi: 10.1371/journal.ppat.1007740; PubMed Central PMCID: PMC6564022. 31194849 PMC6564022

[22] Duran P, Thiergart T, Garrido-Oter R, Agler M, Kemen E, Schulze-Lefert P, et al. Microbial interkingdom interactions in roots promote *Arabidopsis* survival. Cell. 2018;175(4):973–83 e14. Epub 2018 Nov 6. doi: 10.1016/j.cell.2018.10.020 ; PubMed Central PMCID: PMC6218654. 30388454 PMC6218654

[23] Carrow HC, Batachari LE, Chu H. Strain diversity in the microbiome: Lessons from *Bacteroides fragilis*. PLoS Pathog. 2020;16(12):e1009056. Epub 2020 Dec 11. doi: 10.1371/journal.ppat.1009056; PubMed Central PMCID: PMC7728264. 33301530 PMC7728264

[24] Rowley CA, Kendall MM. To B12 or not to B12: Five questions on the role of cobalamin in host-microbial interactions. PLoS Pathog. 2019;15(1):e1007479. Epub 2019 Jan 4. doi: 10.1371/journal.ppat.1007479; PubMed Central PMCID: PMC6317780. 30605490 PMC6317780

[25] Putnam EE, Goodman AL. B vitamin acquisition by gut commensal bacteria. PLoS Pathog. 2020;16(1):e1008208. Epub 2020 Jan 24. doi: 10.1371/journal.ppat.1008208; PubMed Central PMCID: PMC6977713. 31971969 PMC6977713

[26] Iyer N, Vaishnava S. Vitamin A at the interface of host-commensal-pathogen interactions. PLoS Pathog. 2019;15(6):e1007750. Epub 2019 Jun 7. doi: 10.1371/journal.ppat.1007750; PubMed Central PMCID: PMC6553882. 31170262 PMC6553882

[27] Ludington WB, Ja WW. *Drosophila* as a model for the gut microbiome. PLoS Pathog. 2020;16(4):e1008398. Epub 2020 Apr 24. doi: 10.1371/journal.ppat.1008398; PubMed Central PMCID: PMC7179820. 32324814 PMC7179820

[28] Aleman FDD, Valenzano DR. Microbiome evolution during host aging. PLoS Pathog. 2019;15(7):e1007727. Epub 2019 Jul 26. doi: 10.1371/journal.ppat.1007727; PubMed Central PMCID: PMC6657895. 31344129 PMC6657895

[29] Roland MM, Mohammed AD, Kubinak JL. How MHCII signaling promotes benign host-microbiota interactions. PLoS Pathog. 2020;16(6):e1008558. Epub 2020 Jul 1. doi: 10.1371/journal.ppat.1008558; PubMed Central PMCID: PMC7323949. 32598378 PMC7323949

[30] Wilson NG, Hernandez-Leyva A, Kau AL. The ABCs of wheeze: Asthma and bacterial communities. PLoS Pathog. 2019;15(4):e1007645. Epub 2019 Apr 26. doi: 10.1371/journal.ppat.1007645; PubMed Central PMCID: PMC6483249. 31022286 PMC6483249

[31] Litvak Y, Baumler AJ. The founder hypothesis: A basis for microbiota resistance, diversity in taxa carriage, and colonization resistance against pathogens. PLoS Pathog. 2019;15(2):e1007563. Epub 2019 Feb 23. doi: 10.1371/journal.ppat.1007563; PubMed Central PMCID: PMC6383860. 30789972 PMC6383860

[32] Glowacki RWP, Martens EC. In sickness and health: Effects of gut microbial metabolites on human physiology. PLoS Pathog. 2020;16(4):e1008370. Epub 2020 Apr 10. doi: 10.1371/journal.ppat.1008370; PubMed Central PMCID: PMC7144961. 32271839 PMC7144961

[33] Foley MH, O’Flaherty S, Barrangou R, Theriot CM. Bile salt hydrolases: Gatekeepers of bile acid metabolism and host-microbiome crosstalk in the gastrointestinal tract. PLoS Pathog. 2019;15(3):e1007581. Epub 2019 Mar 8. doi: 10.1371/journal.ppat.1007581; PubMed Central PMCID: PMC6405046. 30845232 PMC6405046

[34] Dheilly NM, Martinez Martinez J, Rosario K, Brindley PJ, Fichorova RN, Kaye JZ, et al. Parasite microbiome project: Grand challenges. PLoS Pathog. 2019;15(10):e1008028. Epub 2019 Oct 11. doi: 10.1371/journal.ppat.1008028; PubMed Central PMCID: PMC6786532. 31600339 PMC6786532

[35] Lukes J, Stensvold CR, Jirku-Pomajbikova K, Wegener Parfrey L. Are human intestinal eukaryotes beneficial or commensals? PLoS Pathog. 2015;11(8):e1005039. Epub 2015 Aug 14. doi: 10.1371/journal.ppat.1005039; PubMed Central PMCID: PMC4536199. 26270819 PMC4536199

[36] Mendoza Cavazos C, Knoll LJ. *Entamoeba histolytica*: Five facts about modeling a complex human disease in rodents. PLoS Pathog. 2020;16(11):e1008950. Epub 2020 Nov 13. doi: 10.1371/journal.ppat.1008950; PubMed Central PMCID: PMC7660559. 33180884 PMC7660559

[37] Wang D. 5 challenges in understanding the role of the virome in health and disease. PLoS Pathog. 2020;16(3):e1008318. Epub 2020 Mar 28. doi: 10.1371/journal.ppat.1008318; PubMed Central PMCID: PMC7098563. 32214384 PMC7098563

[38] Duerkop BA. Bacteriophages shift the focus of the mammalian microbiota. PLoS Pathog. 2018;14(10):e1007310. Epub 2018 Oct 26. doi: 10.1371/journal.ppat.1007310; PubMed Central PMCID: PMC6201943. 30359456 PMC6201943

[39] Sullender ME, Baldridge MT. Norovirus interactions with the commensal microbiota. PLoS Pathog. 2018;14(9):e1007183. Epub 2018 Sep 7. doi: 10.1371/journal.ppat.1007183; PubMed Central PMCID: PMC6126856. 30188944 PMC6126856

[40] Neu U, Mainou BA. Virus interactions with bacteria: Partners in the infectious dance. PLoS Pathog. 2020;16(2):e1008234. Epub 2020 Feb 12. doi: 10.1371/journal.ppat.1008234; PubMed Central PMCID: PMC7012391. 32045465 PMC7012391

[41] Knoll LJ, Hogan DA, Leong JM, Heitman J, Condit RC. Pearls collections: What we can learn about infectious disease and cancer. PLoS Pathog. 2018;14(3):e1006915. Epub 2018 Mar 30. doi: 10.1371/journal.ppat.1006915 PubMed Central PMCID: PMC5875890. 29596508 PMC5875890

[42] Butt J, Epplein M. *Helicobacter pylori* and colorectal cancer-A bacterium going abroad? PLoS Pathog. 2019;15(8):e1007861. Epub 2019 Aug 9. doi: 10.1371/journal.ppat.1007861; PubMed Central PMCID: PMC6687094. 31393968 PMC6687094

[43] Ajayi TA, Cantrell S, Spann A, Garman KS. Barrett’s esophagus and esophageal cancer: Links to microbes and the microbiome. PLoS Pathog. 2018;14(12):e1007384. Epub 2018 Dec 21. doi: 10.1371/journal.ppat.1007384; PubMed Central PMCID: PMC6301555. 30571768 PMC6301555

[44] Dheilly NM, Ewald PW, Brindley PJ, Fichorova RN, Thomas F. Parasite-microbe-host interactions and cancer risk. PLoS Pathog. 2019;15(8):e1007912. Epub 2019 Aug 16. doi: 10.1371/journal.ppat.1007912; PubMed Central PMCID: PMC6695093. 31415672 PMC6695093

[45] Lin D, Kouzy R, Abi Jaoude J, Noticewala SS, Delgado Medrano AY, Klopp AH, et al. Microbiome factors in HPV-driven carcinogenesis and cancers. PLoS Pathog. 2020;16(6):e1008524. Epub 2020 Jun 5. doi: 10.1371/journal.ppat.1008524; PubMed Central PMCID: PMC7271998. 32497113 PMC7271998

[46] Kolawole AO, Wobus CE. Gastrointestinal organoid technology advances studies of enteric virus biology. PLoS Pathog. 2020;16(1):e1008212. Epub 2020 Jan 31. doi: 10.1371/journal.ppat.1008212; PubMed Central PMCID: PMC6991956. 31999791 PMC6991956

